# ADEPt, a semantically-enriched pipeline for extracting adverse drug events from free-text electronic health records

**DOI:** 10.1371/journal.pone.0187121

**Published:** 2017-11-09

**Authors:** Ehtesham Iqbal, Robbie Mallah, Daniel Rhodes, Honghan Wu, Alvin Romero, Nynn Chang, Olubanke Dzahini, Chandra Pandey, Matthew Broadbent, Robert Stewart, Richard J. B. Dobson, Zina M. Ibrahim

**Affiliations:** 1 The Department of Biostatistics and Health Informatics, Institute of Psychiatry, Psychology and Neuroscience, King’s College London, De Crespigny Park, London, United Kingdom; 2 Pharmacy Department, South London and Maudsley NHS Foundation Trust, London, United Kingdom; 3 SLaM BioResource for Mental Health, South London and Maudsley NHS Foundation Trust and King’s College London, London, United Kingdom; 4 NIHR Biomedical Research Centre for Mental Health, South London and Maudsley NHS Foundation, London, United Kingdom, Biomedical Research Unit for Dementia, South London and Maudsley NHS Foundation, London, United Kingdom; 5 Department of Health Service & Population Research, Institute of Psychiatry, Psychology and Neuroscience, King’s College London, London, United Kingdom; 6 The Farr Institute of Health Informatics Research, UCL Institute of Health Informatics, University College London, London, United Kingdom; 7 NIHR Biomedical Research Centre, University College London Hospitals, London, United Kingdom; Garvan Institute of Medical Research, AUSTRALIA

## Abstract

Adverse drug events (ADEs) are unintended responses to medical treatment. They can greatly affect a patient’s quality of life and present a substantial burden on healthcare. Although Electronic health records (EHRs) document a wealth of information relating to ADEs, they are frequently stored in the unstructured or semi-structured free-text narrative requiring Natural Language Processing (NLP) techniques to mine the relevant information. Here we present a rule-based ADE detection and classification pipeline built and tested on a large Psychiatric corpus comprising 264k patients using the de-identified EHRs of four UK-based psychiatric hospitals. The pipeline uses characteristics specific to Psychiatric EHRs to guide the annotation process, and distinguishes: a) the temporal value associated with the ADE mention (whether it is historical or present), b) the categorical value of the ADE (whether it is assertive, hypothetical, retrospective or a general discussion) and c) the implicit contextual value where the status of the ADE is deduced from surrounding indicators, rather than explicitly stated. We manually created the rulebase in collaboration with clinicians and pharmacists by studying ADE mentions in various types of clinical notes. We evaluated the open-source **A**dverse **D**rug **E**vent annotation **P**ipeline (ADEPt) using 19 ADEs specific to antipsychotics and antidepressants medication. The ADEs chosen vary in severity, regularity and persistence. The average F-measure and accuracy achieved by our tool across all tested ADEs were 0.83 and 0.83 respectively. In addition to annotation power, the ADEPT pipeline presents an improvement to the state of the art context-discerning algorithm, ConText.

## Introduction

The data available within EHRs is a potentially valuable resource of information describing patient treatment trajectories at low levels of resolution. However, along with the potential lies significant challenges because a notable portion of the EHR is in free-text form, making it necessary to deploy NLP tools to transform the unstructured text into semantically-meaningful annotated knowledge that can be subsequently used to aide in clinical decision making. As a result, the literature contains many efforts to detect and classify clinical named entities in EHR text [1, 2, 3, 4, 5, 6, 7] and resulting in several tools for information extraction from clinical notes including cTAKES [8], MedEx [9] and MetaMap [10]. Recognition of the importance and value of this task has resulted in the creation of a number of challenges by the *Informatics for Integrating Biology and the Bedside Centre* (i2b2) for clinical entity recognition and classification from free-text clinical records [11], including a psychiatry-specific challenge for extracting symptom severity [12].

The tools developed so far have been used to identify a variety of concepts including medications, symptoms, treatments, tests and dosages. Our interest lies in annotating and classifying a specific concept, namely ADEs, which represent troublesome and potentially fatal outcomes of medication treatment and incur substantial burdens on healthcare providers (with projections of annual ADE-related costs approaching £466m) [13]. We focus on annotating and classifying ADEs associated with antipsychotics, and antidepressants medications for two reasons: 1) In psychiatry, many of the factors leading to variations in individual susceptibility to ADEs remain unknown, making the knowledge mined from any potential tool of great value for research and drug evaluation purposes, and 2) Psychiatric EHRs tend to contain most of the ADE-related knowledge in free-text narratives, and therefore require an NLP annotation pipeline for extraction.

The last decade a number of studies have used NLP to identify adverse drug reactions (ADRs; ADEs where a causative relation with medication is established) of interest in free-text EHR documents [14, 15, 16, 17, 18, 19, 20, 21]. However, the NLP tools developed to obtain the results of these efforts have been study-specific, and at times using commercial tools, and therefore neither replicable nor publically available.

Moreover, ADR detection using Psychiatric EHRs exhibit unique challenges that distinguish them from similar tools operating on general hospital EHRs, Adverse Event spontaneous reporting services [22, 23, 24, 25] literature [26] and social media reports [27, 28]. Apart from the well-studied large amounts of redundancy characterising these notes [29], clinical text contains a plethora of hypothetical and retrospective text, historical discussions as well as text negating possible diagnoses and ADRs. This is a direct consequence of the EHRs being filled with not only direct clinical problems, but also detailed summaries of the patient activities, social and family matters, mood, general observations, discussions or warnings given to the patient about potential side effects. Therefore, modelling and identifying the context of an annotation is essential for correct classification.

In our previous work, we developed a rule-based NLP annotation system using manually-created domain expert rules to identify patients who had experienced Extrapyramidal side effects (Dystonia, Parkinsonism, Akathisia, and Tardive Dyskinesia) and adverse drug events (Alopecia, Convulsion, Hypersalivation, Myocarditis, Nausea, Pneumonia and Tachycardia). The system achieved an overall performance of >0.85 precision on these specific ADEs [30]. However, this work focused on identifying patients who had experienced one of the aforementioned ADEs during the course of treatment, rather than identifying all ADE mentions for a given patient and anchoring them to a specific point of time.

In this paper, we extend our previous work to create a rule-based framework which identifies and annotates temporally anchored mentions of all ADEs present in a given clinical text corpus. The modular tool builds on the recommendations for concept extraction and classification resulting from the i2b2 Challenge [11] by 1) identifying ADE mentions, 2) classifying ADE mentions (as positive, implying that the ADE is present, or negative, implying that the ADE is absent) and 3) refining the classification using contextual indicators found in the clinical text.

Our tool comprises a multiphase pipeline targeting ADE-specific patterns in psychiatric clinical text. Our easily expandable dictionary currently houses the vocabulary needed to identify 66 common ADEs representing a comprehensive list of antidepressant and antipsychotic ADRs collated by our lead pharmacy partners whose ADRs are a major interest of ours.

## Methods

### Data source

We acquired data from the Clinical Record Interactive Search (CRIS) [31], a database containing a de-identified replica of the EHRs of four major London, UK-based psychiatric hospitals: 1) The Maudsley Hospital, 2) Bethlem Royal Hospital, 3) Lewisham Hospital and 4) Lambeth Hospital. Conjointly, the four hospitals make up the South London and The Maudsley NHS Foundation Trust, one of the largest mental health provider in Europe serving a population of over 1.2 million patients and storing much of their clinical records and prescribing information in unstructured free text format.

As of January 2017, CRIS contained over 264,000 patient records comprising around 24 million free text documents including correspondence, discharge summaries, events, ward progress notes, mental health care plan and mental state formulations. We extracted 8,321 documents and created 32 corpora, of which around 2,310 documents in four corpora were used for creating the rulebase and remaining around 6,011 documents in 28 corpora for testing, ranging from 130 to 475 documents in each corpus. We created separate corpora for creating the rulebase and testing purposes. The corpus size varies because creating a manual annotation on each mention of ADE is time-consuming and it heavily relied on the availability of clinicians and pharmacist. The size of the corpus left on the expert judgment of clinicians where they agreed the corpus have enough variety of ADEs mention and documents to make a suitable decision. The process we follow as:

1) we extracted all documents within CRIS containing at least one mention of one of 19 ADEs: agitation, akathisia, arrhythmia, galactorrhoea, nausea, myocarditis, cardiomyopathy, constipation, convulsions, diarrhoea, dizziness, dry mouth, hypersalivation, pneumonia, sedation, Steven Johnson syndrome (SJS), tachycardia and weight gain and 2) we further stratified the extracted documents based on the ADE terms mentioned in the document, document length (documents vary in length between a single line and multiple pages) and document types (e.g. discharge summaries, ward progress notes, local GP notes, etc.). The final subset of 8,321 was randomly selected such that the subset contains a variation of document types and lengths for every ADE term.

For creating the rulebase and verification, we manually annotated the 8,321 documents for all mentions of the 19 ADEs in consultation with two clinical and pharmacy researchers who identified and classified mentions of the ADEs. The 19 ADEs were chosen by the clinician and pharmacist represent a range of ADEs ranging from mild to severe, rare to common and short-term to persistent. The selection was based on evidence within the record itself where possible (rare and common), but additionally based on clinical judgement where this was more difficult to derive from the record itself. The level of agreement between the two annotators for all 19 ADEs is given in Table 1 with a percentage representing the agreement and a Cohen’s Kappa scores before removing the 1% documents where the length of the free text was only a single word. In the case of inter-annotator discrepancies, the annotation was reviewed and if ambiguity remained, the document was replaced in the corpus.

**Table 1:** Annotation agreement between two clinical annotators. Annotations were retained as the labelled dataset for predictions if the experts annotators agree on the classifications of their mentions.

ADE	Agreement (%)	Cohen’s Kappa Score
Agitation	88%	0.65
Akathisia	90%	0.75
Arrhythmia	89%	0.73
Cardiomyopathy	89%	0.76
Constipation	91%	0.78
Convulsions	98%	0.96
Diarrhoea	93%	0.84
Dizziness	93%	0.78
Dry Mouth	89%	0.71
Galactorrhea	92%	0.83
Hypersalivation	94%	0.74
Insomnia	92%	0.75
Myocarditis	89%	0.71
Nausea	85%	0.69
Pneumonia	93%	0.82
Sedation	96%	0.91
SJS	93%	0.82
Tachycardia	94%	0.83
Weight Gain	96%	0.90

### Dictionary terms

To accommodate the diversity of writing styles and terminologies used in different hospitals and clinics, and between clinicians and carers, and to account for typographic errors, we compiled a dictionary containing the vocabulary to be used by the different pipeline components. The dictionary also contains variations of ADE-related terms. For example, sedation is a common side effect of antipsychotics. However, depending on context, there are several possibilities to describe sedation such as feeling *sleepy*, *drowsy*, *sleepiness*, *drowsiness*, *sedated*, and *somnolence*. The dictionaries are available for download from our github repository, https://github.com/KHP-Informatics/ADRApp/tree/master/application-resources/ADR.

1. **632 ADE terms**: The ability of the pipeline to recognise and classify additional ADEs is constrained by the ADE terms contained within its dictionary. Our dictionary currently accommodates 66 different ADEs related to antipsychotics and antidepressants, including synonyms and alternate spellings. The vocabulary recogonised by the pipeline is easily extensible with user-provided terms to accommodate additional ADE terms.
2. **2545 drug terms**: derived from the BNF drug dictionary [32], and expanded to include incorrect spellings and updated drug names to reflect coverage within a psychiatric setting (specifically anti-psychotics, anti-depressants, mood stabilisers, hypnotics and anxiolytics).
3. **208 helping terms**: the purpose of which is to indicate ADE occurrences (e.g. ‘does have’, ‘developed’ etc.) and include drug administration (e.g. ‘taking’, ‘applying’, ‘using’, ‘administering’ etc.), monitoring (e.g. ‘assess for’, ‘monitor’, ‘screen for’, ‘signs of’, ‘watch for’ etc.), negative effects (e.g. ‘side effect’, ‘adverse effect’, ‘SE’, ‘EPSE’ etc.) as well as drug link terms (e.g. ‘as it can be’, ‘as it may’, ‘if it cause’, ‘known for’, ‘may lead’ etc.).
4. **660 contextual terms:** these include subject terms (e.g. ‘mother’, ‘patient’), negation terms (e.g. ‘does not’), hypothetical terms (e.g. ‘if’), temporal terms (e.g. ‘previously’) and termination terms (e.g. ‘however’). These terms are partly derived from the 342 terms used in the ConText algorithm [33], an algorithm used for discerning the context surrounding mentions of medical episodes to aid the classification process.

#### Populating the dictionaries

With the help of senior pharmacists, we compiled a list of expected ADRs associated with antipsychotics and antidepressants using the British National Formulary (BNF 68 at the time of research), the electronic medicines compendium (eMC) [34], the Maudsley Prescribing Guidelines 11th Edition and the Micromedex Healthcare database. In addition, a list of possible spelling variations and common alternative descriptions used to describe the ADR in clinical practice was also generated.

### Development environment

We used the GATE NLP framework [35], which is a development environment for creating language engineering applications. GATE offers language-processing, information extraction and testing tools [36]. We used GATE’s Java Annotation Patterns Engine (JAPE) to implement the rule base in all stages of the pipeline [37].

The **A**dverse **D**rug **E**vent annotation **P**ipeline (ADEPt) [38] is composed of four sequentially applied rule-based processing and annotation components. Each component is composed of a set of specialised rules that use co-location and position to correctly annotate ADEs. The overall annotation strategy of the pipeline rests on two observations we made by analysing the structure of the text containing ADE references within the anonymised EHRs:

**Observation 1:** Clinical text usually takes the form of short delimiter-separated clauses with each clause conveying information about a single ADE-related episode. Delimiters are usually periods, commas and semicolons (with periods being the most commonly used). Therefore, we can use delimiter-separated clauses where the annotations are located as boundaries for classification.

**Observation 2:** Clinical text contains a plethora of contextual indicators surrounding ADE mentions including hypothetical and retrospective text, historical discussions as well as text negating possible diagnoses and ADEs. Moreover, ADE-housing clauses can contain multiple contextual indicators. Therefore, a specialised context-discerning component which is capable of resolving conflicts among multiple contextual indicators is necessary to correctly classify ADE mentions.

**Fig 1:**
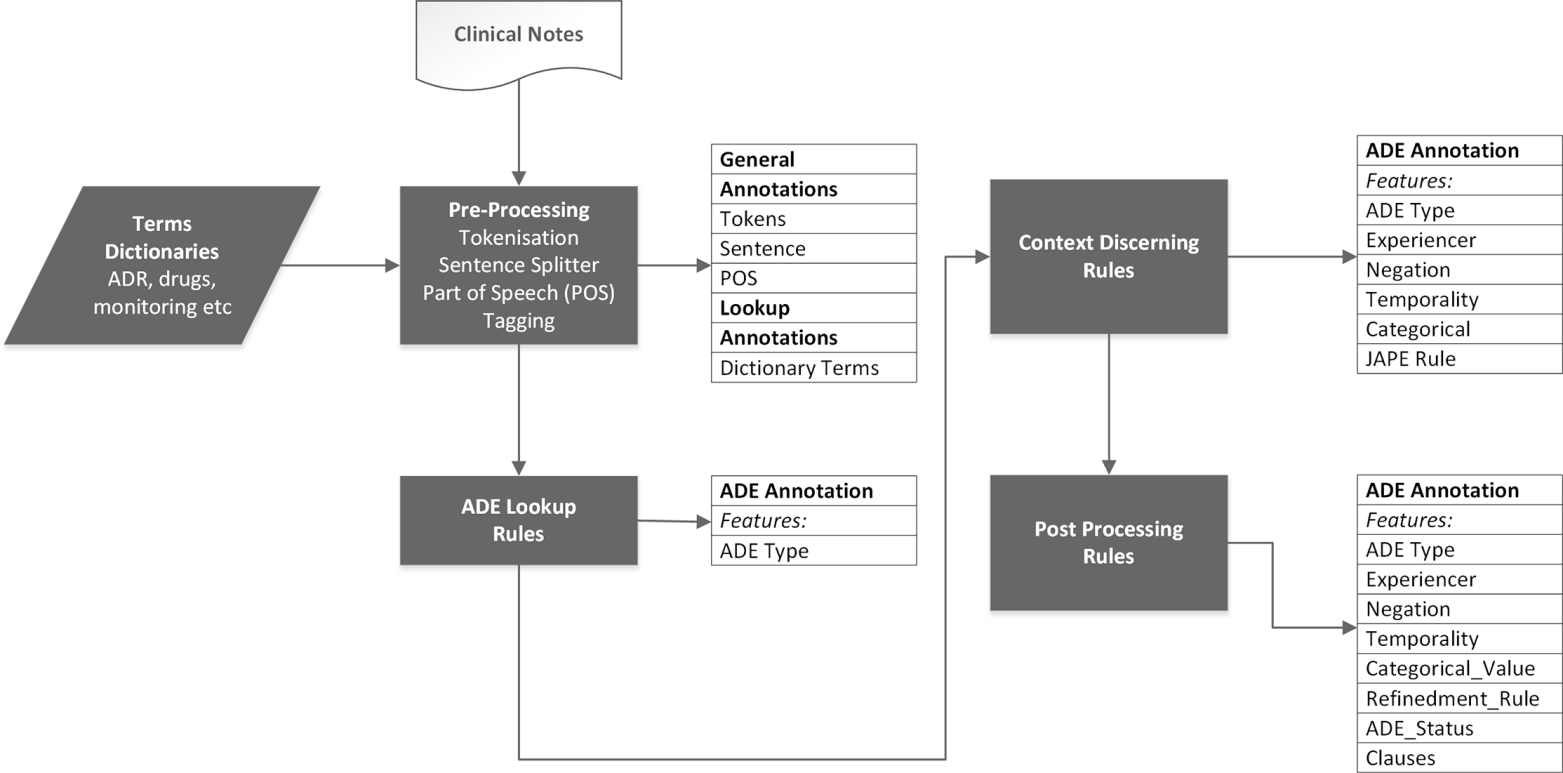
The GATE NLP based ADEPt pipeline comprising four rule-based processing components. The pipeline takes as input EHR clinical notes documents and a dictionary containing all annotation-related terms. The pipeline sequentially applies the four components accumulating new annotations for the target annotation (ADE). The output of the pipeline is a single ADE annotation with six features (ADE type, Experiencer, Negation, Temporality, Categorical_Value, Refinedment_Rule, ADE_status and clause).

A functional representation of the overall pipeline is shown in (Fig 1). ADEPt begins by using the GATE pre-processing resources to prepare an input document for annotation by tokenizing, splitting sentences and tagging parts of speech. For this component, we designed a rule base that specifically examines clause-level boundaries and splits clauses accordingly. The prepared document is then passed to an ADE-identification component which uses the identified boundaries to locate ADE-related terms, as defined in the dictionary, and produce an initial classification (positive or negative).

The third step comprises a set of rules that refine the annotated ADEs by discerning the context surrounding the identified annotation. These rules are triggered when contextual indicators are found in the clauses containing the ADE annotations. We used contextual references to distinguish the subject, the temporal value and the categorical nature of the annotation (i.e. factual, hypothetical or negated) and to identify clauses where the ADE annotations refer to warnings, monitoring, suggestions and ongoing investigations, and implement specialised rules for resolving conflicts among multiple contextual indicators. The pipeline finalises the generated annotations in a post-processing step which attaches metadata summarising the decisions resulting in the final annotation and it’s location in the corpus.

The ADEPt pipeline has two distinguishing features: 1) the use of clauses to delimit the annotation scope and 2) the use of ADE-related text properties to define the context surrounding the identified annotations.

#### Clause-level ADE annotation

Paragraph-level annotation does not offer sufficiently high resolution for our co-occurrence-based annotation system as multiple ADE-containing clauses usually exist in a single paragraph.

**Examples:** The examples below are adapted from real clinical notes of a single patient and are chosen to be representative of the approach we use to extracting ADEs from free text.

1. *Late shift ZZZZZ has wandered in and out of her room throughout the afternoon*. *No complaints of dizziness*.
2. *He did not complain of constipation*. *The patient is still suffering from a light headache*.

The above examples contain multiple-clause paragraphs whereby every clause is delimited by a period (i.e. every clause is a sentence). In the first example, the entire paragraph contains a single ADE mention. Co-location based annotation will examine negation terms co-located with the target ADE within the annotation boundaries and will arrive at the correct decision (a negated mention of dizziness) using both paragraph and sentence-level annotation. This is because the negation term (‘No complaints’) is co-located with the ADE in the same paragraph (and sentence).

On the other hand, the second example shows a paragraph containing two ADE mentions (constipation and headache) in two clauses. If ADE annotation proceeds using paragraph boundaries, the negation term `*did not*’ will lead to the rejection of both ADEs (constipation and headache). This incorrect annotation is also likely when using models based on ‘bag-of-words’ that make decisions dependent on the boundaries defined by the tagging algorithm. Bag-of-word models have in the past led to inconsistent tagging and require complex boundary-detection techniques that our clause-level tagging greatly simplifies. Here, sentence-level tagging will appropriately negate the first ADE (constipation) while affirming the second (headache).

#### The case of multiple ADEs in a single clause

Although clause-level parsing is sufficient in most cases encountered, clinical notes also contain instances of clauses containing multiple ADEs. Manual examination of 8,321 documents showed that approximately 5% of the clauses contained multiple ADE references. Therefore, in order to make correct decisions about ADEs, it is important to accommodate the instances which violate our single-clause, single-ADE assumption.

To approach these situations, we increased the granularity of the annotation process by dividing the clauses containing multiple ADEs into several single-ADE clauses. We did this by creating additional clause-splitting rules to issue splitting actions whenever specific termination terms (as per the dictionary) were present in multi-ADE clauses. For example, the termination term ‘but’ in the clause below divides it into two independent clauses with headache affirmed in the first and constipation negated in the second.

The patient is still complaining of headache but not constipation.

#### Discerning context

We designed a specialised component which uses contextual indicators co-located with the ADE terms to refine the value of the identified annotations. The component comprises a rulebase whose constituents are fired when a context-related trigger term which falls within the scope of an identified annotation is found. For example, the trigger term “no” is associated with a negation context. Once a trigger term is detected within the boundary of the annotation, the context value of the annotation is changed to reflect the value associated with the trigger term.

We built our rulebase by extending the open-source ConText algorithm [33], which identifies negations, hypothetical and general discussions, as well as indicators discerning the temporal validity of the identified annotations (whether they are current or historical). We adapted ConText to our ADE detection context in three ways. First, we customised the algorithm such that is uses the same clause-level boundaries as identified in the previous step. This way, all trigger terms which fall within the same clause as the annotation are captured by the algorithm and are directed to the appropriate rule for classification. Second, although ConText comes bundled with a set of triggering terms, many British English and ADE-relevant terms were not present in its dictionary. Therefore, we added 318 additional terms available in the GitHub repository [33, 38] to ConText’s working dictionary and classified them according to how they affect the identification of an ADE as Table 2 shows. Categories include negation indicators (e.g. ‘excluded’ and ‘not found’), possibility phrases (e.g. ‘most concerned about’ and ‘rule him out for’), experiencer terms (e.g. ‘mother’ and ‘father’), and temporal and hypothetical indicators (e.g. ‘in the past’ and ‘if’). This is in addition to the termination terms discussed in the previous section.

**Table 2:** Enrichment of the ConText algorithm trigger terms.

Triggers Terms	ConText Algorithm (n = 392)	Terms Added (n = 318)	Current Terms (n = 710)
Experiencer	29	46	75
Negation	197	216	413
Possibility terms & phrases	28	16	44
Termination	89	17	106
Temporality & Hypothetical	49	23	72

Finally, although the ConText algorithm is able to correctly classify annotations based on the surrounding context in most instances, there are situations where it is unable to do so. We therefore created an additional set of 26 ‘removal’ and 9 ‘retention’ rules to identify and correctly annotate ADE mentions whose surrounding contextual indicators cannot be properly interpreted by the ConText algorithm. *Retention* rules target annotations that ConText misclassified as negative, and retain them as positive mentions in ADEPT’s results, while *removal* rules target annotations that ConText misclassified as positive, marking them as negative mentions in ADEPt’s final annotations. Removal rules are overridden by retention rules in cases where there is additional evidence that the discussion of the ADE is positive. 26 removal and 9 retention rules are applied to correct the annotation in the following cases:

**1. Retention Rules:** The main aim of retention rules is to identify annotations that are surrounded by negation terms but are nevertheless positive mentions. In the ConText algorithm, a negation term is assigned a high priority and will lead to a negative annotation regardless of the existence of additional terms. However, this overgeneralization fails in many instances in clinical text, as negation may not be used to refer to the patient as given in the examples below:

ZZZZZ appeared to be disorientated and not taking his medication.
ZZZZZ restlessness has not worsened on the increased dose of beta-blocker.

**Fig 2:**
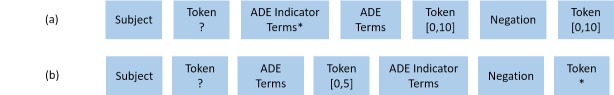
The retention rules pattern used in the ADEPt pipeline.

The corresponding rules are shown in Fig 2(A) and 2(B). In the figure, the term ‘Token’ refers to any token not present in our dictionary.

**Fig 3:**
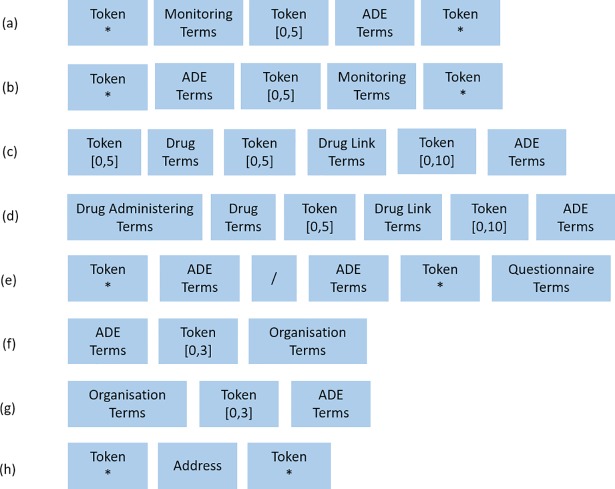
The removal rules pattern used in the GATE NLP based ADEPt pipeline.

**2. Removal Rules:** These rules operate on a number of difference clauses, mainly: a) clauses discussing potential ADEs, ongoing investigations, warning, monitoring or explanations, etc. as in Fig 3(A), 3(B) and 3(C) (e.g. I am changing the dose and have warned ZZZZ of dizziness, or Signs of myocarditis, on going investigation or The patient is starting Olanzapine, explained her as it can cause weight gain), b) clauses where there is uncertainty about whether the ADE is present as in Fig 3(D) (e.g. She has had 4 seizures within the last 2 weeks, unstable partial complex seizures), c) questionnaires, which tend to be pervasive in the electronic text as in Fig 3(E) and 3(F) (e.g. Fainting/ dizziness *No * Yes. I become irritable, restless and nervous x 5) and finally d) ADEs that are part of organisational names or addresses Fig 3(G) and 3(H) (e.g. CENTRE FOR ANXIETY DISORDERS AND TRAUMA, sjs@sydenham.lewisham.sch.uk or www.nhs.uk/Conditions/Anxiety).

#### Ruleset building and testing

From the 8,321documents used in this work, we used 2,310 documents uniformly distributed across the four hospital sites to guide the construction of the rulebase. All documents chosen contain mentions of one of the following four ADEs: Akathisia (common and long-term), galactorrhoea (rare, acute and mild), myocarditis (rare and severe) and nausea (acute and common). The four ADEs chosen for rulebase vary in severity, regularity and persistence. The remaining 6,011 documents were used to test the performance of the pipeline on fourteen ADEs. The ADEs tested include the four used for rulebase in addition to dizziness, hypersalivation, pneumonia, sedation, tachycardia, cardiomyopathy, convulsions, diarrhoea, constipation and Steven Johnson syndrome.

#### Evaluation metrics

We used accuracy, precision, recall and the F-score to evaluate the annotation pipeline (Equations 1, 2, 3 and 4). We also recorded specificity, using it along with recall to examine the shapes of the resulting ROC curves. The metrics rely on true positive (TP), false positive (FP), true negative (TN) and false negative (FN) values, which we defined in terms of the agreement between our pipeline and our annotators consensus for every ADE term found. For example, a true positive annotation is one which, given all associated contextual terms surrounding the annotation, is identified as positive by ADEPt as well as the human annotator. TP is where the subject is the patient, no negation is present and the event is deemed recent. If the match is partial, the ADR is labelled as TN.

### Error analysis

We conducted error analysis at each step of the pipeline to enhance performance. The resulting measures are given in Table 3 and include precision, sensitivity, specificity, accuracy and f-measure. These measures are given for each ADR at the following stages: a) ‘paragraph’, designating the use of paragraphs as delimiters for ADR mentions, b) ‘statement’, designating the use of statements to delimit ADR mentions, c) ‘original ConText Algorithms’ designating the use of the off-the-shelf ConText algorithm without modification, d) ‘original ConText Algorithms’ evaluates the impact of additional vocabulary to the dictionary, and e) ‘With Extra Terms and Refinement Rules’ shows the final performance of the pipeline. Guided by the results obtained at each stage, we a) added more vocabulary to accommodate the unidentified ADRs, and b) added and adjusted the refinement rules to create a more generic rulebase.

**Table 3:** Incremental results of akathisia, galactorrhea, nausea and myocarditis.

ADE	Corpus Ref	Total	Precision	Sensitivity	Specificity	Accuracy	F-measure
Akathisia	Paragraph	215	0.73	0.87	0.33	0.69	0.80
	Statement	215	0.76	0.89	0.39	0.73	0.82
	Original ConText Algorithm	215	0.77	0.90	0.43	0.74	0.83
	ConText With extra terms	215	0.94	0.88	0.88	0.87	0.91
	With Extra Terms and Refinement Rules	215	0.96	0.90	0.93	0.91	0.93
Nausea	Paragraph	369	0.84	0.82	0.52	0.74	0.83
	Statement	369	0.86	0.84	0.56	0.77	0.85
	Original ConText Algorithm	369	0.89	0.87	0.67	0.82	0.88
	ConText With extra terms	369	0.93	0.87	0.80	0.85	0.90
	With Extra Terms and Refinement Rules	369	0.95	0.86	0.84	0.85	0.90
Galactorrhea	Paragraph	139	0.59	0.72	0.41	0.57	0.65
	Statement	139	0.62	0.77	0.45	0.62	0.69
	Original ConText	139	0.66	0.81	0.50	0.66	0.73
	ConText With extra terms	139	0.73	0.89	0.61	0.76	0.80
	With Extra Terms and Refinement Rules	139	0.83	0.91	0.78	0.84	0.87
Myocarditis	Paragraph	188	0.28	0.70	0.30	0.41	0.40
	Statement	188	0.29	0.72	0.32	0.43	0.42
	Original ConText Algorithm	188	0.30	0.74	0.34	0.45	0.43
	ConText With extra terms	188	0.40	0.60	0.64	0.63	0.48
	With Extra Terms and Refinement Rules	188	0.51	0.75	0.71	0.72	0.61

## Results

**Fig 4:**
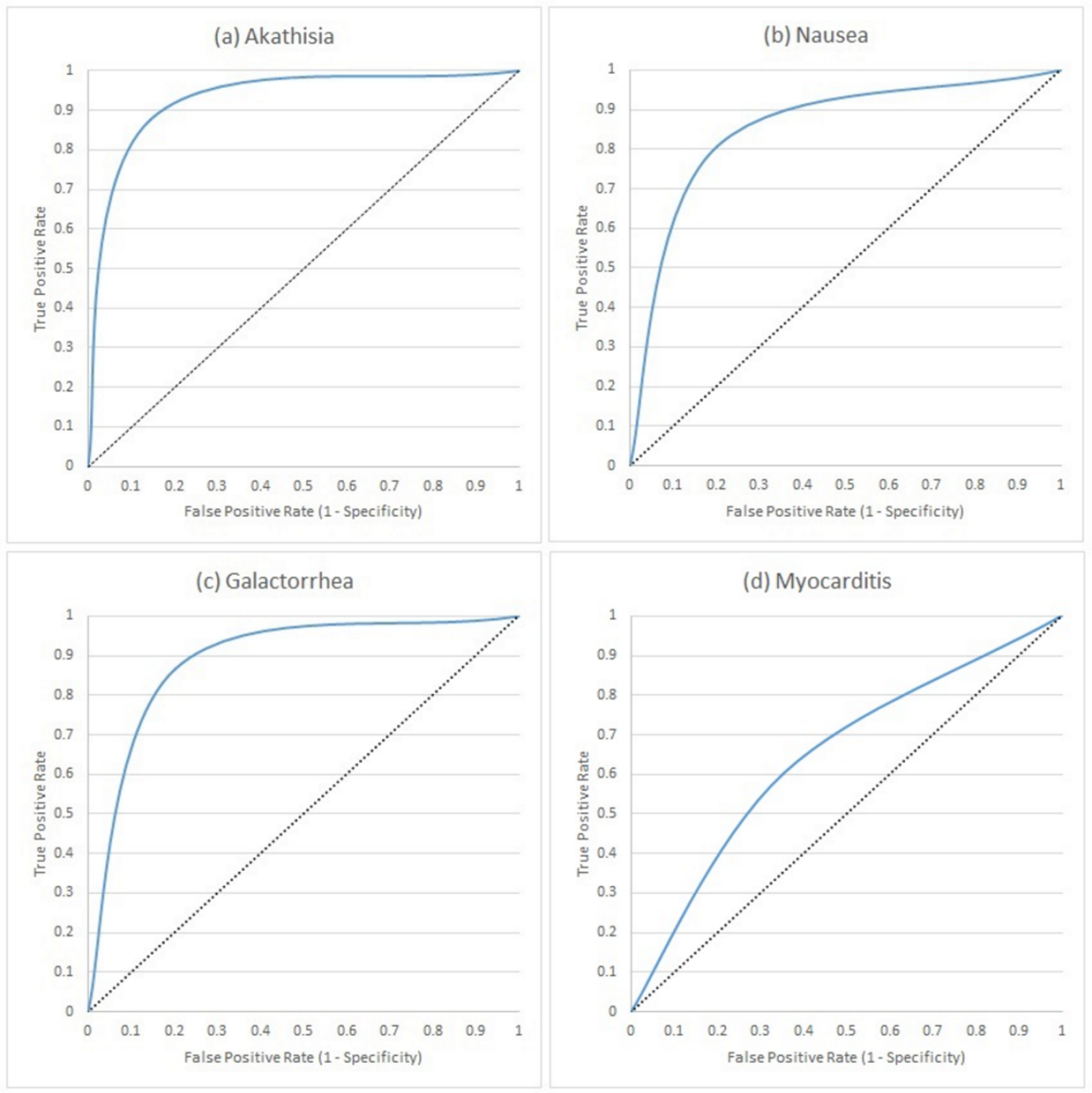
Receiver operating characteristic curves representing the performance of the ADEPt pipeline in identifying akathisia, nausea galactorrhea and myocarditis ADEs from free text. The increments in each graph correspond to 1) our previous work [30], 2) using paragraph boundaries, 3) using clause-boundaries, 4) using unrefined (off-the-shelf) ConText algorithm, 5) adding domain-specific vocabulary to ConText and 6) final refined ConText algorithm.

Here, we present the results of running the pipeline on the manually-annotated 8,321 test set documents for all 19 ADEs. For four ADEs (akathisia, galactorrhoea, nausea and myocarditis), we additionally report the incremental performance increase after each step of the pipeline (Table 3 and Fig 4).

The first and second rows of every ADE in Table 3 show the improvement resulting from using clauses (as opposed to paragraphs) to delimit the annotation scope. Here, we manually examined the TP, TN, FP and FN annotations using clause-level and paragraph-level boundaries and confirmed that: a) none of the correctly-classified ADEs using paragraph boundaries were misclassified using clause boundaries, b) all correctly-classified ADEs using paragraph-level boundaries are proper subsets of those identified by clause-level boundaries.

The last three rows of each ADE show the incremental improvement in classification using contextual indicators found in the text via a) the off-the-shelf ConText algorithm, b) ConText enriched with additional domain-specific terms and finally c) the improved ConText algorithm containing the enriched vocabulary as well as additional refinement rules for conflict resolution and implicit mentions. The incremental improvements are graphically demonstrated using the ROC curves in Fig 4.

Apart from Akathisia, Nausea, Galactorrhea and Myocarditis, we further evaluated ADEPt on fifteen other ADEs (see Table 4). In contrast to our earlier work, the ADEPt pipeline performed well on common ADEs such as constipation, diarrhoea, sedation, hypersalivation, tachycardia, pneumonia, sedation and performed least well in identifying convulsions. ADEPt also performed well on rare ADEs such as cardiomyopathy and Steven Johnson syndrome.

**Table 4:** Results showing the performance of the ADEPt pipeline in identifying a selection of rare to common ADEs related to antipsychotics and antidepressants drugs.

ADE	Total	TP	TN	Precision	Sensitivity	Specificity	Accuracy	F-measure
Agitation	221	142	32	0.89	0.83	0.65	0.79	0.86
Akathisia	215	132	64	0.96	0.90	0.93	0.91	0.93
Arrhythmia	232	129	61	0.88	0.85	0.77	0.82	0.86
Cardiomyopathy	204	55	109	0.79	0.68	0.88	0.80	0.73
Constipation	475	315	99	0.91	0.91	0.76	0.87	0.91
Convulsions	148	84	37	0.92	0.81	0.84	0.82	0.86
Diarrhoea	221	140	55	0.93	0.90	0.83	0.88	0.92
Dizziness	234	130	96	0.94	0.83	0.90	0.85	0.88
Dry Mouth	211	124	56	0.91	0.87	0.82	0.85	0.89
Galactorrhea	139	68	50	0.83	0.91	0.78	0.85	0.87
Hypersalivation	193	161	18	0.97	0.95	0.78	0.93	0.96
Insomnia	189	119	38	0.84	0.93	0.62	0.83	0.88
Myocarditis	188	40	96	0.51	0.75	0.71	0.72	0.61
Nausea	369	241	75	0.95	0.86	0.88	0.90	0.92
Pneumonia	173	81	75	0.89	0.94	0.93	0.90	0.92
Sedation	189	108	54	0.89	0.89	0.81	0.86	0.89
Stephen Johnson’s Syndrome (SJS)	333	68	205	0.60	0.88	0.82	0.82	0.72
Tachycardia	230	192	13	0.96	0.91	0.65	0.89	0.94
Weight Gain	209	108	51	0.92	0.87	0.82	0.86	0.90

The pipeline achieves better performance in common and long-term ADEs than it does with rare and acute ADEs. This directly reflects the number of mentions of the corresponding ADEs in the clinical notes, which affects the number of patterns detected upon manual examination of the 2,310 documents we used to guide the creation of the rules. This variation in performance is also visible upon examining the ROC curves of Fig 4. A final observation from Table 4 is that some rare ADEs (SJS and cardiomyopathy) appear to have more mentions in the clinical notes than common ADEs (when examining the second column of the table), which may appear counterintuitive. However, this increased count is explained by the fact that these two ADEs are severe, driving clinicians to document any warnings or monitoring performed for them, and resulting a large number of negative mentions of these ADEs (as evident by the third column).

## Discussion

We created a multi-phase rule-based pipeline for the recognition and classification of named ADEs in free-text psychiatric EHRs. The rulebase was created by manually analysing 2,310 of these documents in collaboration with domain experts to identify patterns of ADE mentions and related contextual text. We constructed the rulebase based on four ADEs (akathisia, nausea, myocarditis and galactorrhea) and evaluated its performance using these four ADEs as well as 15 ADEs related to antipsychotics and antidepressants drugs in 6,011 unseen clinical text documents by comparing with manual annotations by clinical researchers.

The clinical text contains many surface forms for any ADE mention, necessitating a unifying dictionary to guide the annotation ADE process. In collaboration with pharmacists and clinicians based at the South London and Maudsley NHS Foundation Trust, London, United Kingdom, we compiled a list of 66 ADEs with corresponding surface forms, misspellings and abbreviations. In addition to compiling agreed-upon medical terms from existing resources, the process involved large-scale manual examination as many ADE-related terms are implied rather than explicitly defined, e.g. a clinician may record ‘the patient cannot fit in his/her clothes’ in lieu of explicitly documenting weight gain.

Overall, the tool performs well compared to general NLP entity recognition systems, specifically the Context algorithm which we used for comparison. However, the performance varied depending on the regularity and persistence of the ADE under investigation. There is still a need to improve the context discerning rules for rare ADEs, which are usually discussed as possibilities in the clinical notes as clinicians usually take a lot of care before ruling out the possibility of a rare ADE and will administer multiple tests for the patients to go through. The current application does not have a high coverage for all the precautionary patterns surrounding multiple ADEs, an issue to address in our current work.

The annotations generated by the tool were compared to the manually-annotated documents prepared by domain experts. In this work, we only trained and tested ADEPt using annotations where the expert annotators agree on the classification of the ADE mention (i.e. whether it is a positive or a negative mention). It would be interesting to see whether the annotations that confused the human annotators (ones where the two experts disagree) will similarly confuse ADEPt. Therefore, part of our ongoing work is to add a third classification category corresponding to ambiguous annotations.

Additional ongoing work includes improving the context-discerning rules to distinguish rare from common ADEs. Moreover, we are investigating the merit of a hybrid approach which learns the rules and examine the effect on performance. Our ongoing work also focuses on linking ADE annotations obtained through ADEPt with medication and prescription information, to create a timeline establishing the associations between ADEs and medication episodes as well as possible drug-drug interactions.

All of the ongoing efforts aim at using the annotated knowledge mined by ADEPt to uncover unknown causal links between administered medications and the undesirable events, therefore making the distinction between ADEs we mine from the clinical text and non-preventable Adverse Drug Reactions (ADRs) which are caused by the medication itself and not due to mismanagement or clinical errors [39, 40].

### Limitation

There are a few aspects of the ADEPt pipeline on which we are currently working. One of the issues we faced since the inception of our work is the difficulty of finding experts to annotate the documents and evaluate the annotation results against the annotated documents. As a result, the annotation power of ADEPt has only been evaluated on the 19 ADRs mentioned throughout the manuscript. However, we tried to minimise the effect of the small number of ADRs by selecting those which vary in terms of severity as well as frequency of onset in order to reflect the variations of mentions in the clinical text. Another consequence of the difficulty of finding annotators is that we have used four different annotators throughout the different stages of pipeline development, which may have induced unknown discrepancies in some of the cases.

Another limitation of the ADEPt pipeline is due to the limited number of clinical notes discussing the onset of rare ADEs such as SJS and myocarditis. Due to their rarity (as well as severity), discussions of these ADEs is usually done in the context of potential onset (negative mentions), rather than positive mentions referring to the patient herself. However, for these ADEs, ADEPt achieves better and both precision and sensitivity have improved in SJS simultaneously (0.60 and 0.88) and sensitivity in myocarditis (0.75).

Finally, ADEPt is developed and tested on SLaM’s psychiatric clinical notes. Work evaluating its performance on other general or psychiatric notes is currently part of our ongoing work.

## Conclusion

The tool described here demonstrates the ability to identify antipsychotics and antidepressants related ADEs from within the free text of psychiatric EHRs. By surfacing ADEs within the routinely collected EHR, we unlock a treasure trove of hitherto inaccessible data describing treatment response that is the first step to tailoring treatment through patient stratification leading to opportunities for novel interventions and studies into the genetic underpinnings of ADEs. The tool is freely available from our online repository at: https://github.com/KHP-Informatics/ADRApp
